# More Negative FRN From Stopping Searches Too Late Than Too Early: An ERP Study

**DOI:** 10.3389/fnins.2021.705000

**Published:** 2021-09-06

**Authors:** Mei Gao, Xiaolan Yang, Linanzi Zhang, Qingguo Ma

**Affiliations:** ^1^School of Business and Management, Shanghai International Studies University, Shanghai, China; ^2^School of Business Administration, Guizhou University of Finance and Economics, Guiyang, China; ^3^Institute of Neuromanagement Science, Zhejiang University of Technology, Hangzhou, China; ^4^School of Management, Zhejiang University, Hangzhou, China

**Keywords:** search behavior, regret, ERPs, feedback, gender, risk attitude

## Abstract

It is widely known that the feedback from a decision outcome may evoke emotions like regret, which results from a comparison between the gain the decision-maker has made and the gain he/she might make. Less is known about how search behavior is linked to feedback in a sequential search task such as searching for jobs, employees, prices, investments, disinvestments, or other items. What are the neural responses once subjects decide to stop searching and receive the feedback that they stopped too early or too late compared with the optimal stopping time? In an experimental setting of a search task, we found that the feedback-related negativity (FRN) induced by the feedback from stopping too late was more negative than stopping too early, suggesting that subjects might experience stronger regret when stopping too late. Subjects preferred to stop searching earlier if the last feedback was that they stopped too late, and vice versa, although they did not always benefit more from such adjustment. This might reflect general patterns of human learning behavior, which also manifests in many other decisions. Gender differences and risk attitudes were also considered in the study.

## Introduction

Since people are often confronted with dynamic choice problems in their daily lives, search behavior has long been a topic of considerable interest in job searches (McCall, 1970; Burdett, 1978; Cox and Oaxaca, 1989; Le Barbanchon et al., 2021), information searches (Punj and Staelin, 1983; Brucks, 1985), and price searches to exercise an option or terminate an investment (Ihlanfeldt and Mayock, 2012; Yang et al., 2018).

Previous studies pay lots of attention to the search duration and reservation value of search behavior. Individuals preferred to stop search earlier than the optimal search duration derived from the rational risk-neutral assumption (Schunk and Winter, 2009). Viefers (2012) found that ambiguity-averse decision-makers reacted to ambiguity by postponing the investment relative to a situation where there was a risk. In search tasks, the reservation value is the least favorable point at which one will accept to stop searching. Asano et al. (2015) designed a laboratory experiment to explore the effect of ambiguity on subjects’ search behavior. They observed that subjects reduced their reservation points in the face of ambiguity over point distribution. In a real-time-search laboratory experiment, subjects’ reservation wages declined sharply over time. However, in the widely accepted labor market search models, the payoff-maximizing reservation wage was constant (Brown et al., 2011).

The feedback of decisions is essential since people tend to compare the actual benefit from “what is” with “what might have been.” The feedback about actual and foregone outcomes might induce emotions like regret as a motivation for decisions (Humphrey, 2004). Regret comes from an inner source when the outcome of a decision is worse than the other decision. What’s more, feedback has a significant effect on individual behavior in terms of the search strategy. Individuals were likely to deviate from the optimal strategy (Sonnemans, 2000), but they could learn from the search outcomes and make adjustments. For example, if they observed that shortening search durations might yield a higher payoff, they would stop searching earlier in subsequent searches to adjust this behavior (Einav, 2005).

Literature has identified an ERP component, feedback-related negativity (FRN), that may be especially relevant to feedback-driven emotion like regret (Luo et al., 2011). The FRN peaks approximately 200–300 ms following feedback, especially indicating negative compared to positive feedback. Moser and Simons (2009) suggested that the FRN reflected a context-sensitive signal that integrated information about current and past actions, thoughts, and emotions. They interpreted their results to mean that the FRN should be largest on trials in which regret is largest. FRN is more pronounced for negative feedback associated with unfavorable outcomes than for positive feedback. Search outcomes are always negative when compared with the best payoff. People seldom make optimal search decisions, and their search outcome is worse than the best outcome generated from the optimal search decision. Therefore, the FRN is suited to investigate the neural mechanism of feedback in search behavior.

Unlike the static choice problems, the feedback in search behavior compares the benefit from the chosen option with the benefit from the alternative options. It shows whether individuals stop search too late or too early compared with the optimal stopping time to earn the best payoff. The highest price in a trial would yield the best payoff. Therefore, there are two kinds of feedback in a sequential search task by comparing the stop time with the timing of the highest price. First, subjects stop searching after the appearance of the highest price (hereafter, “stop searches too late”), and second, subjects stop searching before the arrival of the highest price (hereafter, “stop searches too early”). Which scenario induces a more negative FRN? This question has not been discussed in the literature.

Based on the previous findings, there are some differences between “stop searches too late” and “stop searches too early.” Sonnemans (1998) believed that subjects who stopped early had less opportunity to learn than subjects who stopped late because the difference in the amount of information gathered by the subjects caused a difference in learning opportunity. Seale and Rapoport (2000) found stopping too early was more costly than stopping too late. What’s more, some psychology studies consider two forms of regret, namely action and inaction regret. Inaction was associated with greater short-term regret than action (Gilovich and Medvec, 1995; Abendroth and Diehl, 2006; McElroy and Dowd, 2007). In our experiment, the feedback of “stop searches too late” was inaction as subjects had already seen the highest price, but they did not take action to sell the stock at the timing of the highest price. The feedback of “stop searches too early” was an action. Therefore, we were interested in exploring whether subjects would have different neural responses like FRN, which probably had a close relationship with regret, when stopping searches too late and too early.

To test our hypothesis concerning the modulation of the FRN by different kinds of feedback, we designed a sequential search task composed of 50 trials including different kinds of feedback described above. The stock price was randomly drawn from a uniform distribution in each trial and presented to subjects in each of the 20 periods. Subjects were asked to decide when to sell their stocks within a trial. In this trial, once subjects stopped searching to sell their stocks at a price, feedback about the highest price was immediately presented to the subjects. Their event-related potentials (ERPs) were recorded.

In this study, there were three types of feedback when comparing the optimal stopping time at which the highest payoff yielded with subjects’ own stopping time: the subjects stop searches too late, too early, or optimally. We also compared the subjects’ average search duration and ERPs. Risk attitudes and gender differences were considered in our study.

## Materials and Methods

### Subjects

Twenty-three students in Zhejiang University from different majors were recruited via an advertisement posted on BBS. There were 22 valid data for analysis (7 females, mean age = 23.00 ± 2.29). One subject’s data was rejected because his data was not completely recorded due to the loose contact of the device. All of the subjects were right-handed with normal or corrected-to-normal vision. The show-up fee of the experiment was CNY20 (around 3.1 dollars). An additional monetary reward was associated with subjects’ performance. In terms of the exchange rate with actual earnings, 100 tokens in the experiment equaled CNY1. Informed consent was obtained in writing before the experiment, and the subjects were paid for their participation after the experiment. The study was approved by the Ethics Committee, Neuromanagement Lab, Zhejiang University.

### Experimental Design

#### The Search Task

**FIGURE 1:**
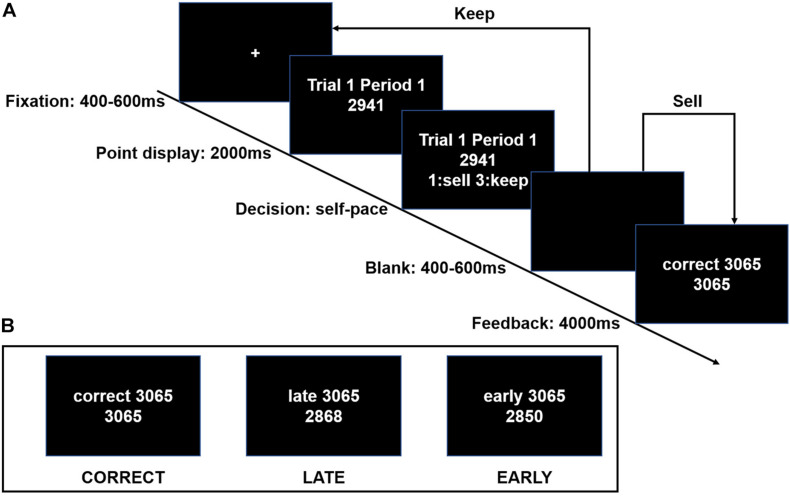
Experimental design. **(A)** Single-trial settings. Each trial began with a fixation in the center of the screen. Within each trial, subjects decided when to sell their stocks in 20 periods. Once the subject decided to sell his/her stocks at the current price, the searching task was finished, and feedback was presented. If the subject rejected to stop (keep), he/she returned to the fixation of the next period. They had to sell the stocks in a limited 20 periods in each trial. **(B)** Three examples of feedback.

We designed a sequential search task (Figure 1) to investigate how search behavior was linked to the feedback in a dynamic search task, and the difference between the degrees of the emotion of regret triggered once subjects received the feedback about whether they stopped searches too early or too late, compared with the optimal stopping time that yielded the highest payoff.

The task consisted of two blocks of 25 trials each. The subjects were endowed with a stock portfolio at a fixed cost before each trial. Each trial began with a fixation in the center of the screen. Within each trial, the subjects who acted as investors decided when to sell their stocks across 20 periods. The price of the stock portfolio was randomly drawn from a uniform distribution from 2,000 to 4,000 and was presented on the screen at the beginning of each period. All the prices had been randomly generated by the computer before the experiment and were the same for all subjects. Once the subject decided to sell his/her stocks at the current price, the search task was finished, and the information about the highest price in this trial was presented. Finally, this trial was concluded, and the accepted price of the stock portfolio was converted into a payment. If the investor rejected to stop (keep), then he/she returned to the fixation of the next period. The subject had to sell the stocks in a limited 20 periods in each trial.

As Figure 1 shows, three examples of outcome feedback presentation in the final block. Comparing the optimal stopping time that yielded the highest payoff with subjects’ own stopping time, subjects were, respectively informed of “correct,” “late,” and “early.” In a particular trial, for example, if subjects sold their stocks at period 12 while the highest price had appeared at period 9 or was not presented until period 15, then they were informed of their having stopped “late” or “early,” respectively, by the presented feedback in the final block. In the first line, the number 3065 represents the highest price of the stock portfolio in Trial 1, while the number in the second line represents the subjects’ accepted price.

Feedback was given once subjects stop searching in one trial. There were three types of feedback comparing the optimal stopping time, yielding the highest payoff, with the subjects’ own stopping time, that is, “CORRECT,” “LATE,” and “EARLY.” CORRECT indicated that subjects sold their stock at the highest price; LATE indicated that they sold their stock after the highest price appeared (the subjects stopped searches too late); EARLY indicated that they sold their stock before the time the highest price appeared (the subjects stopped searches too early).

The subjects used a numpad to choose to sell or keep the portfolio of stocks. Pressing number 1 meant “sell” while pressing number 3 meant “keep.” The search task was repeated across 50 trials. Only one trial was randomly selected to determine the real payoff for the subjects.

#### Procedure

The experiment was conducted with the software E-Prime. Before the experiment, all of the subjects were asked to wash and blow-dry their hair. They then seated themselves comfortably on a chair that was one meter away from a 17-inch CRT screen in an acoustically isolated and electrically shielded room. After a public reading of the instructions, two trials were conducted to facilitate subjects to practice the search task. Then, the first block of the search task was started. The trials were conducted one at a time. After the first block of the search task, the subjects were reminded to sit comfortably and rest for 5 min. When the rest time was finished, the second block of the search task was started. At the end of the search task, we used a lottery choice task (Holt and Laury, 2002) shown in Table 1 that was modified from previous studies to measure the risk attitude of the subjects, which consisted of 10 paired lottery choices between a safe option and a risky option. Each subject’s degree of risk aversion was measured by the number of safe options chosen by him/her.

**TABLE 1:** The 10-paired lottery choice task by Holt and Laury (2002).

**No.**	**Option A**	**Option B**	**Expected payoff difference**
1	1/10 of CNY 2.00, 9/10 of CNY 1.60	1/10 of CNY 3.85, 9/10 of CNY 0.10	CNY 1.17
2	2/10 of CNY 2.00, 8/10 of CNY 1.60	2/10 of CNY 3.85, 8/10 of CNY 0.10	CNY 0.83
3	3/10 of CNY 2.00, 7/10 of CNY 1.60	3/10 of CNY 3.85, 7/10 of CNY 0.10	CNY 0.50
4	4/10 of CNY 2.00, 6/10 of CNY 1.60	4/10 of CNY 3.85, 6/10 of CNY 0.10	CNY 0.16
5	5/10 of CNY 2.00, 5/10 of CNY 1.60	5/10 of CNY 3.85, 5/10 of CNY 0.10	–CNY 0.18
6	6/10 of CNY 2.00, 4/10 of CNY 1.60	6/10 of CNY 3.85, 4/10 of CNY 0.10	–CNY 0.51
7	7/10 of CNY 2.00, 3/10 of CNY 1.60	7/10 of CNY 3.85, 3/10 of CNY 0.10	–CNY 0.85
8	8/10 of CNY 2.00, 2/10 of CNY 1.60	8/10 of CNY 3.85, 2/10 of CNY 0.10	–CNY 1.18
9	9/10 of CNY 2.00, 1/10 of CNY 1.60	9/10 of CNY 3.85, 1/10 of CNY 0.10	–CNY 1.52
10	10/10 of CNY 2.00, 0/10 of CNY 1.60	10/10 of CNY 3.85, 0/10 of CNY 0.10	–CNY 1.85

### ERP Recordings

We used a 64-channel ERP system (Scan 4.3, Neurosoft Labs, Inc.) to record EEG with electrodes mounted according to the extended international 10/20 system during the experiment. The raw EEG and EOG data were low-pass filtered with a cut-off frequency at 30 Hz (24 dB/Octave), and then re-referenced to the average of the left and right mastoids. Eye blinks and movements were recorded from left supraorbital and infraorbital electrodes, while the horizontal EEG was recorded from electrodes placed 1.5 cm laterally to the left and right external canthi. All electrode impedance was maintained below 5 kΩ. The raw EEG data were visually inspected for artifacts in an off-line analysis first. Eye movement artifacts were corrected with an ocular artifact correction algorithm provided by Neuroscan 4.3 software. Next, the EEG data was segmented from −200 to 800 ms relative to the target onset. All of the trials in which EEG voltages exceeded a threshold of ±80 μV during the recording epoch were excluded from averaging. The EEG recordings for every subject were separated into two kinds of feedback, LATE feedback and EARLY feedback.

### Data Analysis

The Mann–Whitney statistical method was a non-parametric test and was always adopted to analyze the behavioral data. We compared the different average search duration in this trial after the reveal of the LATE and EARLY feedback. We also examined the effect of gender and risk attitude on the search behavior of subjects. Random-effects generalized least squares regression was used to further explore the concrete degree of the feedback type’s effect on search duration.

**FIGURE 2:**
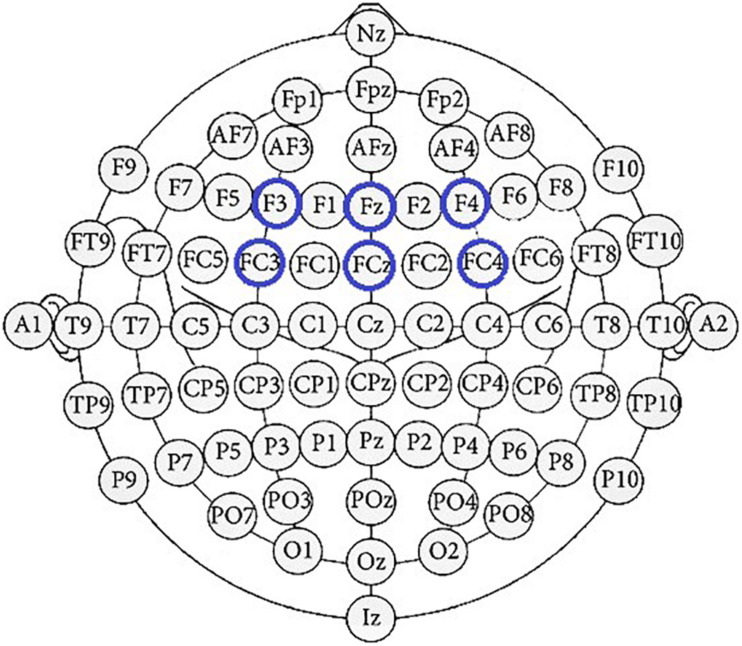
The extended 10–20 International system of EEG electrode placement. Blue circles represent the channels of feedback-related negativity (FRN).

Repeated measures analysis of variance (ANOVA) was adopted to perform a statistical analysis of ERP results. In this study, the dependent variable of ANOVA was the amplitudes of FRN, and the independent variables were feedback with two types (LATE vs. EARLY feedback) and electrodes with six levels (F3, Fz, F4, FC3, FCz, FC4). As Figure 2 shown, we focus on these six electrodes on which the FRN was great (Leng and Zhou, 2010; Zheng et al., 2017; Yaple et al., 2018).

## Results

### Behavioral Results

**FIGURE 3:**
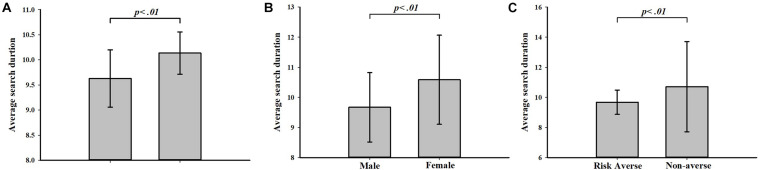
Average search duration (+5% standard error) under different conditions. **(A)** Average search duration under two types of feedback. **(B)** Average search duration of male and female subjects. **(C)** Average search duration of risk-averse subjects and non-averse (risk-seeking and risk-neutral) subjects. Risk aversion increases with the number of choosing safe options out of the 10 paired choices. The subjects were clarified as risk-averse if the number of safe options was more than 4 and non-averse if the number of safe options was equal to or less than 4.

From the average perspective, subjects’ search behavior was affected by the different feedback types of searches, gender, and risk attitude. Our analysis focused on the LATE and EARLY trials rather than the CORRECT trials because almost no subject could perfectly stop searches “correctly.” The direct comparison showed different search durations in this trial after revealing different feedback in the last trial (Figure 3A). The average search duration across all of the trials after the LATE feedback was significantly shorter than after the EARLY feedback (mean ± SD, 9.12 ± 2.10 vs. 10.46 ± 1.99, Mann–Whitney test, *p* = 0.000). We also found that female subjects tended to stop later than male subjects (10.61 ± 5.05 vs. 9.67 ± 5.29, Mann-Whitney test, *p* = 0.0046; Figure 3B), which was in line with the previous study (Ibanez et al., 2009). Further analysis also indicated that the risk-averse subjects stopped earlier than the non-averse subjects, including risk-seeking and risk-neutral subjects (9.70 ± 5.05 vs. 10.71 ± 5.64, Mann–Whitney test, *p* = 0.0093; Figure 3C).

We also used random-effects generalized least squares regression to further examine the effect of feedback type on subjects’ search duration and the effect of gender and risk attitude. The regression results are displayed in Table 2. The coefficients of LATE and RiskAverse are significantly negative and the coefficients of Female are significantly positive in column (1) without control variables and in column (2) with control variables. In line with the result of the Mann–Whitney test, taking the regression result in column (2) for example, the duration under LATE feedback is significantly 0.74 shorter than the duration under EARLY feedback. Furthermore, female subjects’ search duration is 1.37 longer than male subjects’ search duration and risk-averse subjects’ search duration is 1.14 shorter than not risk-averse subjects’ search duration. The regression in column (3) of Table 2 is to test whether gender and risk attitude moderate the effect of feedback type on search duration through the interaction of feedback type and gender/risk attitude. The coefficient of LATE becomes not significant since more variables are added to the regression. The coefficients of the interaction terms are not significant, which shows that the moderating effect of gender and attitude doesn’t exist.

**TABLE 2:** The regression results.

	**Dependent variable: Search duration**		
**Independent variables**	**(1)**	**(2)**	**(3)**
LATE	−0.66* (0.36)	−0.74* (0.39)	−0.21 (0.71)
Female	1.31*** (0.39)	1.37*** (0.39)	1.55*** (0.50)
RiskAverse	−1.11*** (0.40)	−1.14*** (0.39)	−0.95* (0.51)
LATE × Female			−0.51 (0.78)
LATE × RiskAverse			−0.50 (0.81)
Constant	10.60*** (0.37)	9.30*** (1.85)	9.24*** (1.85)
Control variables	No	Yes	Yes
R square	0.0932	0.1123	0.1248
Observations	933	933	933

***1% significance level.

*10% significance level.

*Standard error is in the parentheses.*

*Dependent variable: Each subject’s search duration in every trial.*

*Independent variable:*

*LATE: = 1 if last trial’s feedback is “LATE”; = 0 if last trial’s feedback is “EARLY.”*

*RiskAverse: = 1 if subjects choose more than four Option A in the lottery choice task.*

*LATE × Female: Interaction of variable *LATE* and *Female*.*

*LATE × RiskAverse: Interaction of variable *LATE* and *RiskAverse*.*

*Control variables: Age and OptimalDeviation are control variables.*

*OptimalDeviation is defined as the difference between the price yielding highest payoff and the price that subjects sell at in the last trial.*

*“Yes” means these control variables are included in the regression, and “No” means these control variables are not included in the regression.*

### ERP Results

**FIGURE 4:**
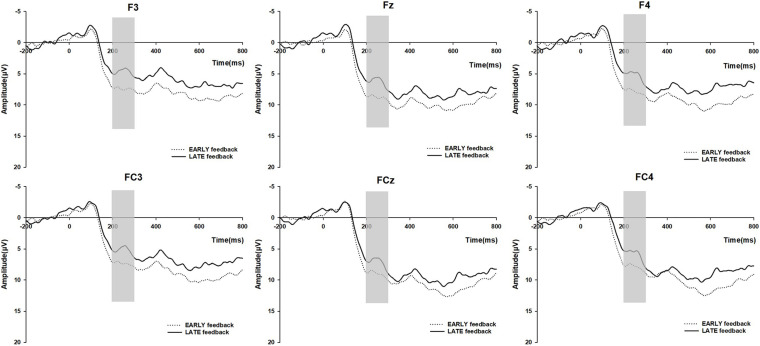
Grand average ERP waves for EARLY feedback/LATE feedback trials at the F3, Fz, F4, FC3, FCz, and FC4.

For the FRN analysis, we measured the average amplitude in the 200–300 ms time window after feedback onset (Falkenstein et al., 2000; Gehring and Willoughby, 2002). Figure 4 shows the grand average wave elicited by LATE and EARLY feedback at the F3, Fz, F4, FC3, FCz, and FC4. Here, a 2 (feedback type: LATE, EARLY) × 6 (Electrodes: F3, Fz, F4, FC3, FCz, FC4) repeated measures ANOVA on FRN amplitudes depicted the significant main effect of feedback type [*F*(1,21) = 16.747, *p* = 0.001, $\eta_{p}^{2}$ = 0.444]. The amplitude of FRN triggered by the LATE feedback (mean ± SD, 5.71 ± 0.74) was significantly more negative than that under the EARLY feedback (mean ± SD, 8.05 ± 0.99). Therefore, we supposed that the more negative amplitude of FRN might indicate a stronger experience of regret (Moser and Simons, 2009; Luo et al., 2011; Qi et al., 2011).

**FIGURE 5:**
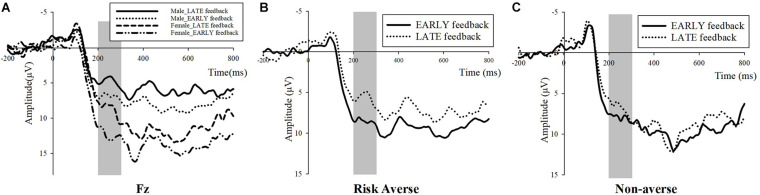
**(A)** Gender difference in FRN amplitude (7 females, 15 males). **(B)** FRN amplitude under two types of feedback among risk-averse subjects (16 risk-averse subjects, 6 risk-neutral and risk-seeking subjects). **(C)** FRN amplitude under two kinds of feedback among non-averse (risk-seeking and risk-neutral) subjects.

To test the gender differences in the amplitude of FRN, we employed a repeated measures ANOVA with feedback type (LATE, EARLY), Electrodes (F3, Fz, F4, FC3, FCz, FC4), and gender (male, female) in which gender is a within-subject factor. The main effect of feedback was still significant, *F*(1, 20) = 18.01, *p* < 0.001, $\eta_{p}^{2}$ = 0.474. Importantly, there was also a significant main effect of gender, *F*(1, 20) = 5.99, *p* = 0.024, $\eta_{p}^{2}$ = 0.230. Male subjects had a more negative FRN response to both LATE feedback and EARLY feedback than female subjects, as Figure 5A shows. However, the interaction between feedback and gender did not reach significance (*p* > 0.1, $\eta {}_{p}^{2}$ = 0.059).

Risk-averse subjects and non-averse subjects displayed considerably different patterns in the waves of FRN (Figures 5B,C, repeated measures ANOVA with feedback type (LATE, EARLY) and Electrodes (F3, Fz, F4, FC3, FCz, FC4), *F*(1,15) = 20.607, *p* < 0.001, $\eta_{p}^{2}$ = 0.579, for risk-averse subjects; *F*(1, 5) = 0.701, *p* > 0.1, $\eta_{p}^{2}$ = 0.123, for non-averse subjects). The repeated measures ANOVA on FRN amplitudes revealed a significant main effect of feedback type only among risk-averse subjects.

### Feedback-Driven Adjustment

Post-decision regret, elicited by the reveal of feedback indicating more profit from an alternative decision, is the driver of learning (Ert and Erev, 2007; Marchiori and Warglien, 2008). In general, a decision-maker adopts a feedback-driven adjustment (Einav, 2005). Thus, in our experimental setting, we assumed that subjects would shorten their search duration in this trial after receiving the LATE feedback and increase their search duration after receiving the EARLY feedback.

**FIGURE 6:**
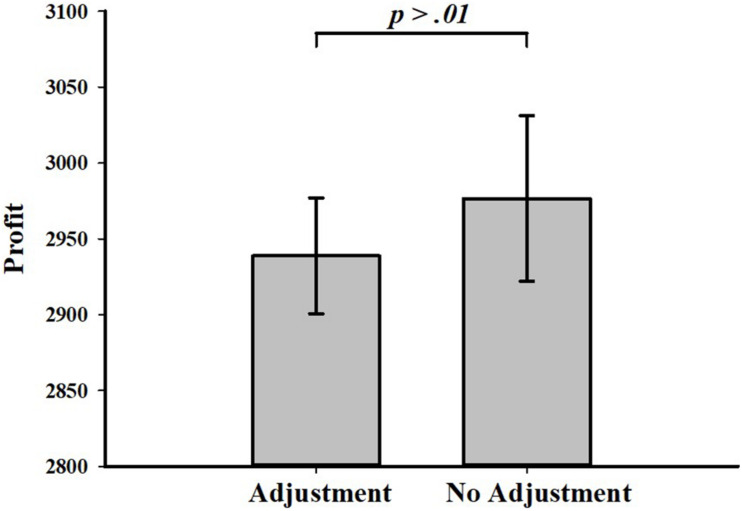
Profit made by using and not using feedback-driven adjustment.

Most of the subjects (68.98% overall trials) were observed to adjust their search duration in this trial according to the feedback from the last trial. Obviously, the subjects preferred to learn from failures and adopt feedback-driven adjustments in their search decisions. However, Figure 6 illustrates that there is no difference between the profit made from trials in which subjects adjusted search duration according to feedback and trials in which subjects did not do so (Mann-Whitney test, *p* = 0.339). Subjects tended to adjust their decisions in the search task, even though they were uncertain whether or not the profit made by adjusting would be higher.

## Discussion

The present study aimed to determine whether the LATE feedback (stop searching too late) or EARLY feedback (stop searching too early) induced a more negative FRN which probably reflected stronger feelings of regret. ERP data demonstrated that compared with the optimal stopping time yielding the highest payoff, people had a more negative FRN when stopping searching too late than too early. One possible explanation for the difference in FRN amplitude between the LATE feedback and EARLY feedback was that people might feel much more regret when they found that they had stopped searching too late than too early. Moreover, such a type of regret could drive people to adjust their search behavior. Specifically, in this trial, subjects stopped searching earlier after receiving the LATE feedback and stopped searching later after receiving the EARLY feedback.

Understanding the neural basis of choice in optimal stopping problems is of fundamental importance because it relates to many problems of economic decision-making (Houser et al., 2004; Viefers, 2012). Our paper is the first to investigate the neural response to feedback in a dynamic search framework to the best of our knowledge. The difference between FRN amplitude under two types of feedback suggested that subjects might experience different degrees of regret when they stopped their searches too late and too early.

Regret plays a vital role in decision-making (Connolly and Zeelenberg, 2002; Humphrey, 2004). Subjects were inclined to make regret-minimizing choices rather than risk-minimizing choices (Zeelenberg et al., 1996). It was reasonable that increased regret aversion might lead to more careful decisions (Reb, 2008). In our study, the emotion of regret may arise once subjects decide to stop searching and receive the feedback that they stopped too early or too late compared with the optimal stopping time. In the search task, the decision is irreversible, and there is only one opportunity to decide at a certain time. The future payoff of a decision is also uncertain. The characteristics of such decisions can evoke the experience of regret after a reveal of outcomes by which people benefit less from “what is” compared with “what might have been” (Bell, 1982; Sugden, 1985; Roese and Olson, 2014). Regret theory assumed that the utility of a chosen option additionally depended on the feelings evoked by the outcome of the rejected option (Loomes and Sugden, 1982). Individuals might experience regret because they may reflect on how much better their position would have been if they had chosen differently. This reflection may reduce the psychological experience of pleasure driven from the outcome they had chosen. However, few studies focus on emotion triggered from the revelation of the search results that may affect the subsequent search behavior.

We found that the possible neural signal of regret, the FRN amplitude, was more negative under LATE feedback than that under EARLY feedback, perhaps because subjects felt more regret over selling stocks too late rather than too early. One possible explanation relates to uncertainty; with the increase of uncertainty, the value of investment decreases (Nishimura and Ozaki, 2007). Because uncertainty is resolved when stopping a search, when the subjects were informed that they stopped earlier than the optimal stopping time, their experience of regret was compensated by the elimination of future uncertainty.

Learning driven by regret-based feedback may predict individual behavior (Ert and Erev, 2007; Marchiori and Warglien, 2008). Our results showed that regret might be triggered by the comparison between the possible highest payoff and their payoff in the sequential search task had a significant effect on the search behavior of subjects. Apparently, subjects usually adjusted their search strategy in this trial just after receiving feedback from the last trial. This might reflect general human behavior, and it is similar to what is witnessed in economic settings (Einav, 2005), organizational operations (Chuang and Baum, 2003), and surgery units within medical facilities (Kc et al., 2013). However, in our experiment, such simple learning might not have always brought more profit because, in complex financial markets, stock prices could follow random paths and were difficult to predict.

In sum, our study used ERP and a sequential search task to reveal different neural responses, the FRN, after receiving different types of feedback. These findings suggest that subjects may experience stronger regret when they stopped searching too late. Although subjects did not always benefit from adjustments, they tried to act in the opposite direction of the feedback and expected to stop their search task at a better time that was closer to the optimal stopping time. This might reflect human learning behavior, and it helps us to better understand individual search behavior, which also exists in many other aspects of human life. There are two limitations of this study. First, we did not measure subjects’ feelings of regret by an emotion rating scale. Second, regret is a complex emotion related to many ERPs, but our paper focused on the FRN. In future studies, we will add the emotion rating scale to the experiment and explore the component processes underlying regret, such as P3, Pe and so on.

## Data Availability Statement

The raw data supporting the conclusions of this article will be made available by the authors, without undue reservation.

## Ethics Statement

The studies involving human participants were reviewed and approved by The Neuromanagement Laboratory Ethics Committee at Zhejiang University. The patients/participants provided their written informed consent to participate in this study.

## Author Contributions

XY, MG, LZ, and QM designed the experiment, wrote and revised the manuscript, and finally approved the version to be published. MG and LZ performed the experiment. XY and MG analyzed the data. LZ and QM drew the figures. All authors contributed to the article and approved the submitted version.

## Conflict of Interest

The authors declare that the research was conducted in the absence of any commercial or financial relationships that could be construed as a potential conflict of interest.

## Publisher’s Note

All claims expressed in this article are solely those of the authors and do not necessarily represent those of their affiliated organizations, or those of the publisher, the editors and the reviewers. Any product that may be evaluated in this article, or claim that may be made by its manufacturer, is not guaranteed or endorsed by the publisher.
